# Genome-wide localization of the polyphenol quercetin in human monocytes

**DOI:** 10.1186/s12864-019-5966-9

**Published:** 2019-07-23

**Authors:** Dana Atrahimovich, Avraham O. Samson, Yifthah Barsheshet, Jacob Vaya, Soliman Khatib, Eli Reuveni

**Affiliations:** 1 Department of Oxidative Stress and Human Diseases, MIGAL – Galilee Research Institute, 11016 Kiryat Shmona, Israel; 2 Faculty of Medicine in the Galilee, Bar-Ilan University, 1311502 Safed, Israel; 3 Tel-Hai College, 12208 Upper Galilee, Israel

**Keywords:** Polyphenol, Quercetin, Chem-seq, RNA-seq, Anticancer

## Abstract

**Background:**

Quercetin is a polyphenol of great interest given its antioxidant activity and involvement in the immune response. Although quercetin has been well studied at the molecular level as a gene regulator and an activator of specific cellular pathways, not much attention has been given to its mechanism of action at the genome-wide level. The present study aims to characterize quercetin’s interaction with cellular DNA and to show its subsequent effect on downstream transcription.

**Results:**

Two massive parallel DNA-sequencing technologies were used: Chem-seq and RNA-seq. We demonstrate that upon binding to DNA or genome-associated proteins, quercetin acts as a cis-regulatory transcription factor for the expression of genes that are involved in the cell cycle, differentiation and development.

**Conclusions:**

Such findings could provide new and important insights into the mechanisms by which the dietary polyphenol quercetin influences cellular functions.

**Electronic supplementary material:**

The online version of this article (10.1186/s12864-019-5966-9) contains supplementary material, which is available to authorized users.

## Background

Quercetin is a major polyphenol, known for its anti-inflammatory, neuroprotective, cardioprotective, and chemopreventive activity [1, 2]. Quercetin is ubiquitous in fruit, vegetables, teas and wines, as well as in dietary supplements. For many years, quercetin’s mode of action was assumed to stem from its antioxidant properties—donating electrons or chelating transition metals. However, antioxidant activity is not the sole explanation for quercetin’s cellular effects in vivo, since it is poorly absorbed, extensively metabolized, and its blood concentrations barely reach the level needed for effective antioxidant activity [3]. Recent studies have suggested that polyphenols’ mode of action is mediated by different, not yet fully explored mechanisms, such as interaction with proteins and nucleic acids [4]. Polyphenols in general, and quercetin in particular, readily penetrate cellular and nuclear membranes and accumulate in the cell nucleus [5]. Polyphenols have been shown to interact selectively with different components of protein kinases and alter their phosphorylation state, thus regulating multiple cell-signaling pathways [6–8]. Polyphenols can also interact with, and modulate the endogenous activities of estrogen receptors, thereby slowing or even preventing the development of breast and ovarian cancers [8, 9]. In addition, polyphenols have been found to interact with various transcription factors and to regulate cell-cycle arrest, apoptosis and survival [10]. Polyphenols have been shown to interact with enzymes (e.g., hydrolases, oxidases, kinases), altering their structure and activity [11, 12]. Similarly, they have been found to act as ligands of nuclear receptors, modulating homeostasis [3]. Recent studies in our laboratory have found that polyphenols bind specifically to plasma proteins and lipoprotein particles and modify the lipoprotein’s physical and biological structure. In another study, we found that quercetin binds to an allosteric site of the serum enzyme paraoxonase 1, affecting its function and biology [11]. Although polyphenols are known to regulate gene expression and modulate signal-transduction pathways, the precise mechanisms remain unclear [13]. Experiments in the last 3 years have suggested that polyphenols bind to specific DNA-sequence motifs; for example, quercetin interacts with the dodecamer duplex sequence, whose structure has been solved (PDB ID: 1BNA) [14]. Recently, the structure of quercetin complexed with c-myc G-quadruplex DNA was solved by nuclear magnetic resonance (NMR) spectroscopy [15]. While the interactions of some polyphenols, such as quercetin, resveratrol, genistein and curcumin with DNA are known, the mode of binding, the precise location of polyphenol-binding sites on the DNA, and their function at the genome level have not been investigated [16].

Today, next generation sequencing (NGS) technologies can provide the complete genome of an organism following an established laboratory protocol. Moreover, it is possible to extract DNA from specific regions by cell manipulation and then obtain its nucleotide sequence. One of the first applications of NGS was ChIP-seq (chromatin immunoprecipitation followed by sequencing). Recently, a new NGS application has been reported that allows extracting and sequencing DNA regions bound to small chemical molecules, namely Chem–seq (chemical affinity capture and massive parallel DNA sequencing). In contrast to ChIP-seq, which targets DNA regions by using a specific antibody against a known chromatin, Chem-seq allows capturing chromatin regions bound to small molecules without prior information, using an unbiased, nonspecific marker such as biotin [17]. Biotin can be integrated into the molecule of interest, and then streptavidin can selectively filter only biotinylated molecules bound to chromatin regions which can then be mapped to the genome. Although Chem-seq is a newly emerging NGS application, several reports have already illustrated its ability to isolate known drug–chromatin interactions [17, 18]. In the present study, Chem-seq and RNA-seq were performed, and their data analyzed and combined to gain unique insights into the interaction of quercetin molecules with the genome. We show that quercetin binds to monocytes’ chromatin and acts as a cis-regulatory element. In particular, we show that quercetin modulates the expression of genes involved in the cell cycle and cell development. Our findings provide novel insights into the mechanism by which the polyphenol quercetin affects monocyte biology.

## Results

### Biotinylated quercetin synthesis

Previous studies have shown that both the physicochemical properties and bioactivities of polyphenols can be significantly improved via conjugation with cleavable promoieties, such as pivaloxymethyl (POM) or isopropyloxycarbonylmethoxy (POC) groups, to its 7-OH position [19]. Based on these observations, we prepared quercetin that was biotinylated at position 7-OH (Additional file 1: Figure S1a). The biotinylated quercetin adduct was analyzed by high-performance liquid chromatography (HPLC) and liquid chromatography–mass spectrometry (LC–MS) (Additional file 1: Figure S1b), and found to correspond to the expected mass.

### Localization of quercetin genomic binding sites using Chem-seq

To identify the genome-wide binding activity of quercetin in human monocyte cell line THP 1, we used the Chem-seq technique schematically described in Fig. 2b to determine whether quercetin is associated with regulatory elements, as we hypothesized, we analyzed our Chem-seq results using peak-finding algorithms. We used HOMER [20] and MACS2 [21] for the sequence reads obtained from bead and quercetin–bead DNA libraries and selected significantly overlapping peak calls resulting from both algorithms. To exclude potential bead signals from the quercetin treatment, we excluded overlapping peak calls from beads and quercetin. Our final list contained 44,000 significant bead peaks and 1,400 quercetin peaks. For downstream analysis, we chose the most significant 1,000 peaks from each list. We used GREAT [22] for enrichment analysis of quercetin and bead peaks up to 500 bp upstream of the transcription start site (TSS) and found that 36 genes from the quercetin peak list corresponded to the cell cycle and cell division (Fig. 2c, Table 1). In contrast to the significant enrichment of genes found in the quercetin treatment, the equivalent 1,000 bead peaks did not result in any functional enrichment. To check for a potential DNA motif site for those 1,000 peaks, we used the HOMER de novo motif algorithm to scan for motifs in our dataset and compare them with known HOMER motifs. Corresponding with the gene-enrichment results, we detected significant E2F DNA motif enrichment in the quercetin treatment (Fig. 2d). E2F is a gene family that encodes transcription factors involved in cell-cycle regulation [23]. Importantly, no significant enrichment was found in the beads-only treatment.

**Table 1:**
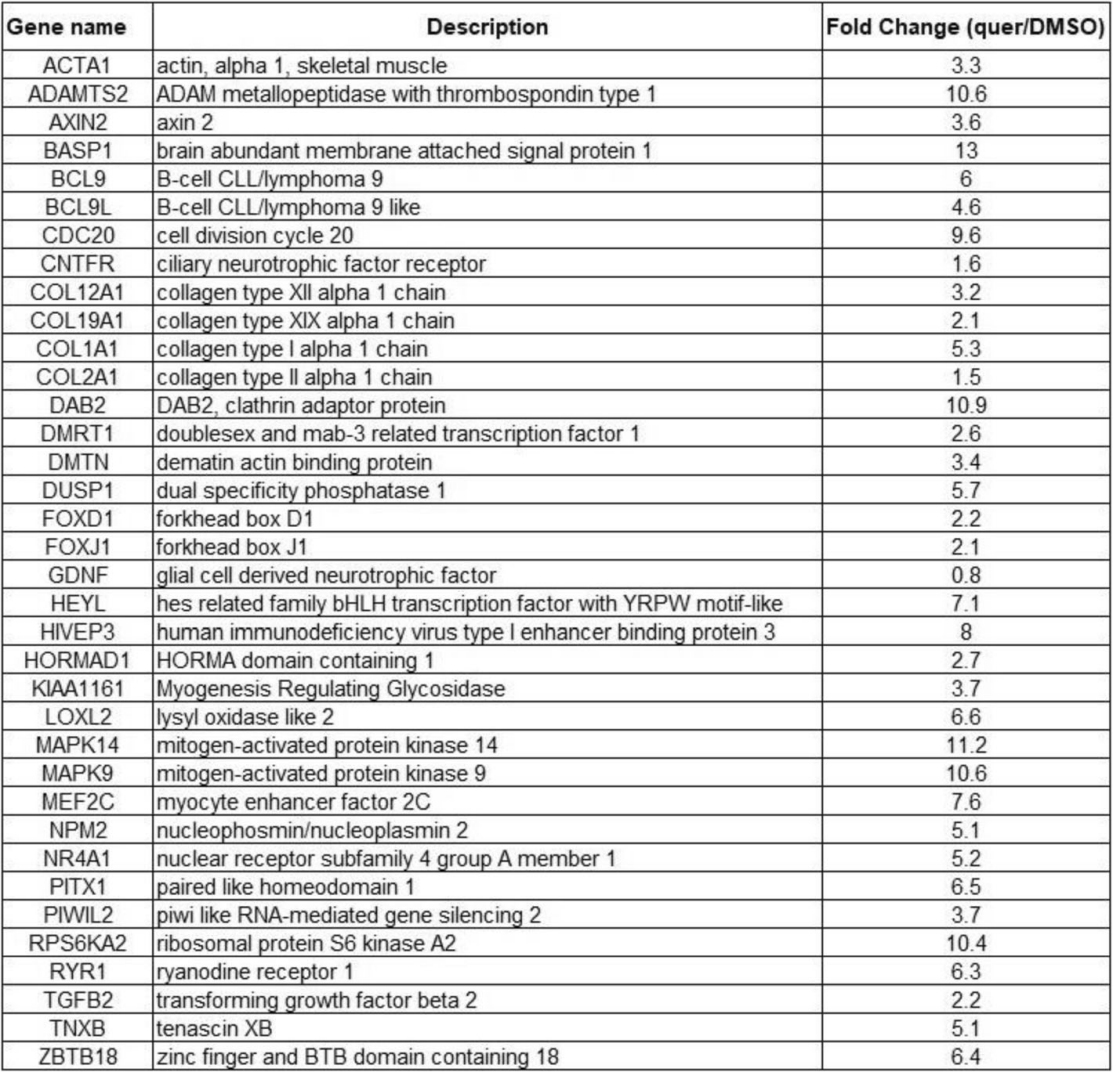
List of the 36 cell cycle genes and fold change (FC) after Quercetin treatment. It is notable that Quercetin induces over-expression as direct regulator as illustrated by the chem-seq results

### Quercetin alters cis-regulatory genes in monocytes

To determine whether quercetin affects transcriptional reprogramming in cis, we performed RNA-seq for quercetin and dimethylsulfoxide (DMSO) treatments (see Methods), resulting in 16,308 coding genes, of which 1,869 were found to have significant differential expression with fold change (FC) between 0.5 and 2 and false discovery rate (FDR) < 0.05. Quercetin is known to have a global effect on transcription, either directly or through modulators [10, 13]. The promotor enrichment results displayed ~ 36 cell-cycle genes with peaks at ~ 500 bp from the TSS (Table 1). We assume that these genes are under direct quercetin regulation. We selected the 36 genes to test for significant FC in gene expression levels between quercetin (treatment) and DMSO (control). Comparing the FC values of those 36 genes with the FC levels of the background showed that quercetin tends to increase (*P* = 0.02) expression (median = 1.2) compared to the background (median = 1) (Fig. 3b).

Although our results indicated transcriptional reprogramming of quercetin, we wanted to verify that this was not obtained by pure chance. To exclude randomality, we used a permutation test of the FC values of the 36 empirical genes compared to a randomly selected group of genes (Fig. 3a). The permutation test was performed as follows: 10 cycles of 100 permutations were run. In each cycle, we compared the empirical FC values to the FC of a random set of 36 genes using the Kolmogorov–Smirnov test and recorded the *P* values (denoted as *P*empirical). To exclude reproducibility of the test, we used the Kolmogorov–Smirnov test between two random selections of 36 genes and recorded the *P* values (denoted as *P*random). Then, we compared the distribution of *P*empirical and *P*random values (Fig. 3a) and found that the former tends to be lower than the latter. This means that the FC values of the 36 genes are likely to be significantly different from those of a random set. In contrast, the distribution of *P*random values (obtained from 36 FC values of two random sets) was higher, suggesting that selection of two random sets of genes does not give significant FC values. Moreover, while the 10 permutation cycles produced a uniform distribution shape for *P*empirical, *P*random tended toward higher values with nonuniform distribution. These data supported our hypothesis that cell-cycle gene expression is altered after quercetin induction.

### Biotin marker does not affect expression profile among the 36 genes

We compared the effect of biotinylated quercetin (Fig. 2a) on gene expression with that of quercetin. We prepared another RNA-seq assay with monocyte cells, except that this time we used the biotinylated quercetin as the treatment and DMSO as the control and measured the FC values. The FC values of the 36 genes under quercetin and biotinylated quercetin regulation were similar (*P* = 0.4, *t* test), but the latter were significantly different from the background gene expression (*P* < 1e^− 15^, *t* test). Our analysis thus showed that quercetin and biotinylated quercetin have similar effects on gene expression.

## Discussion

**Fig. 1:**
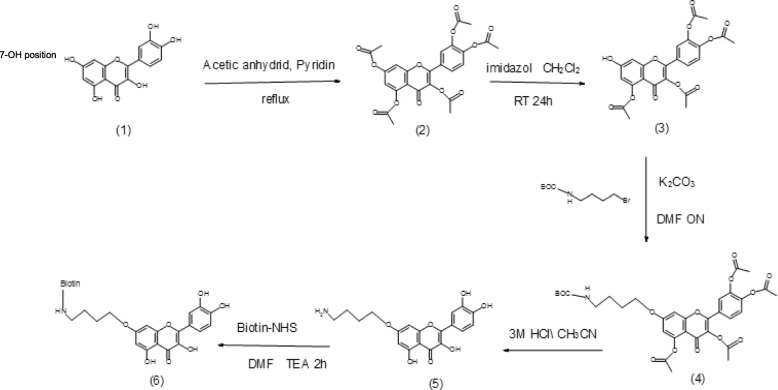
Synthesis of biotinylated quercetin. The biotinylated quercetin was synthesized in 5 steps (see Methods), and compounds are labeled from 1 to 6

**Fig. 2:**
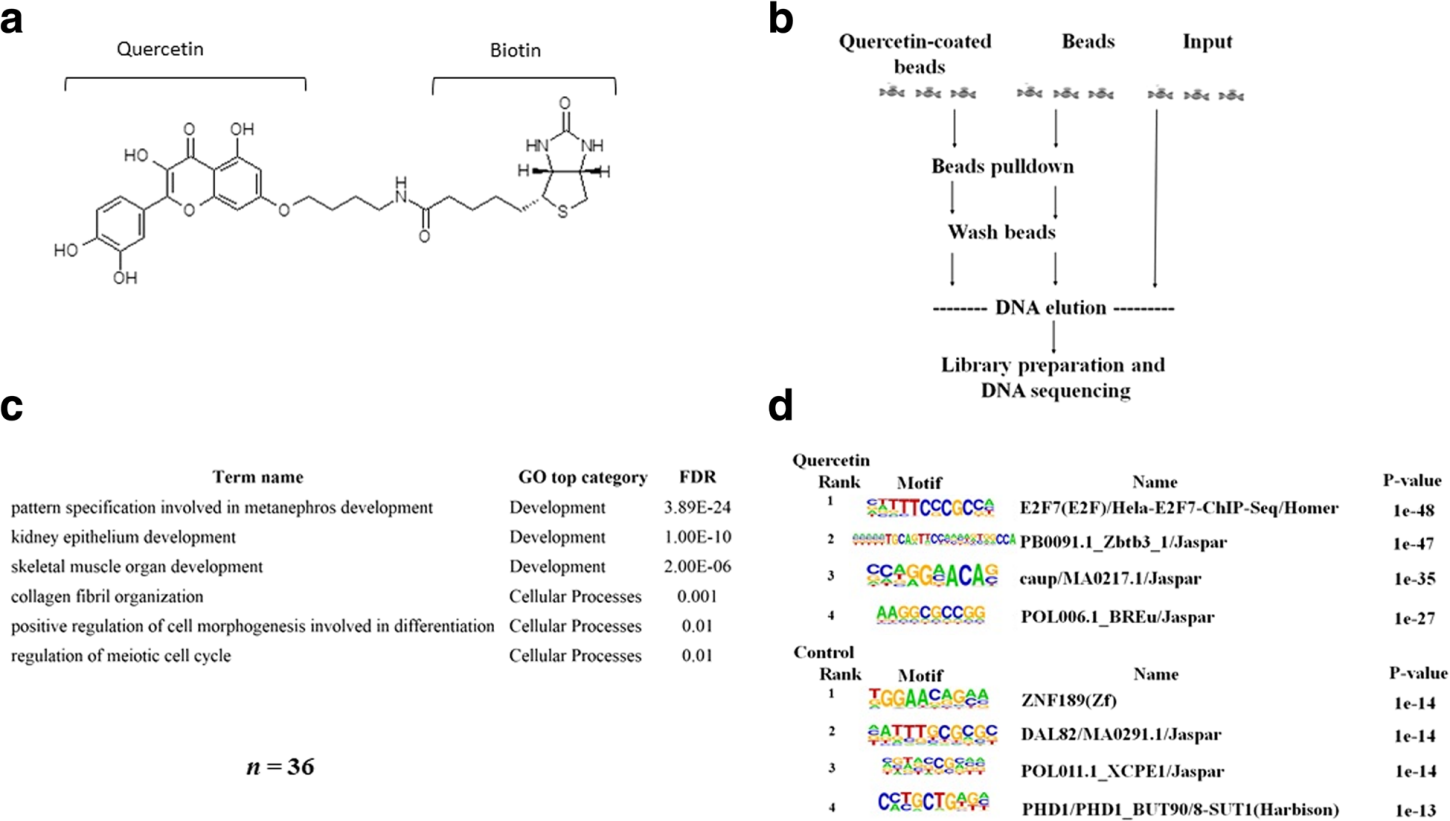
Colocalization of quercetin beads with DNA and the 1,000 most significant peaks across the genome. **a** Structure of biotinylated quercetin product. **b** Flowchart of Chem-seq procedure. **c** Gene ontology (GO) enrichment of the 36 genes associated to the quercetin peaks up to 500 bp from the transcription start site. Bead peaks did not tend to cluster into any functional category. **d** Motif enrichment in quercetin and bead (control) treatments. Bead motif enrichment did not result in any significant motif hit

**Fig. 3:**
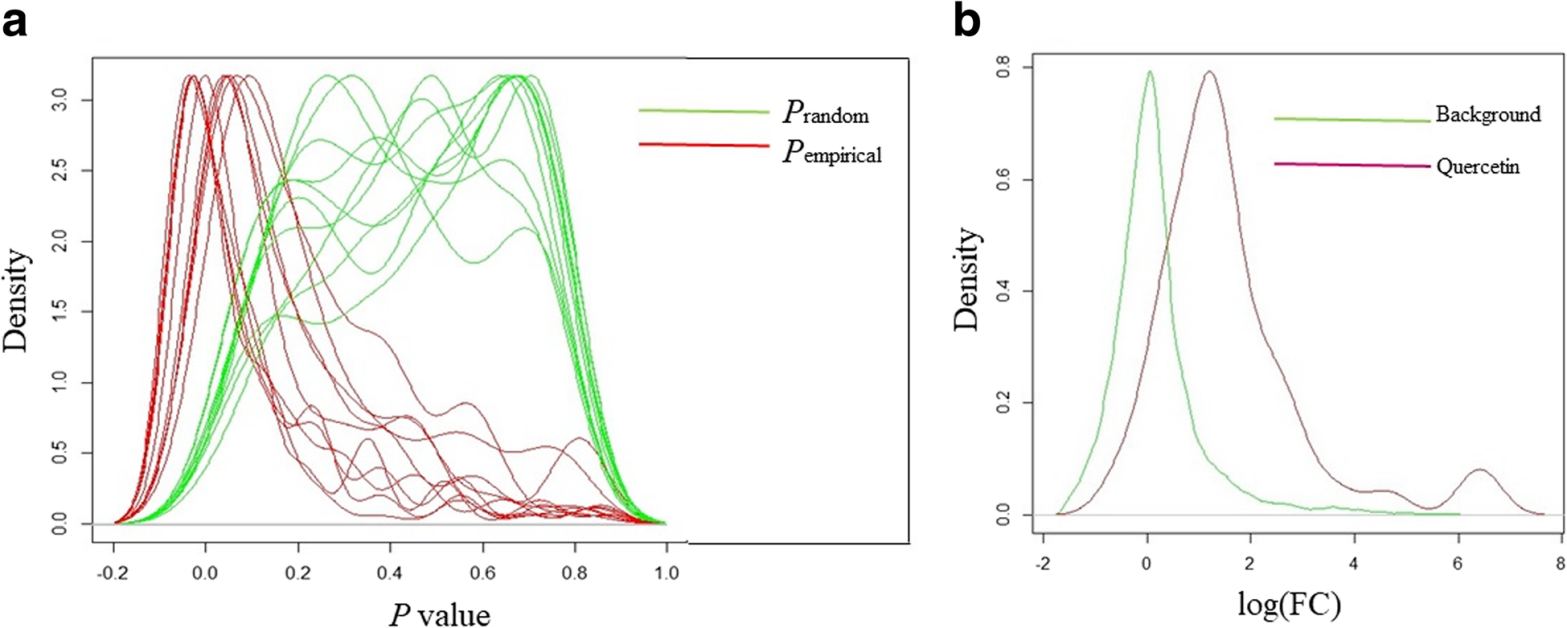
Distribution of *P* values and FC values of gene expression before and after quercetin treatment. **a** Kernel density estimates of *P* values obtained from 10 cycles of 100 permutations. *P* value was obtained by using Kolmogorov–Smirnov test between two sets of 36 FC values. *P*random denotes the distribution of *P* values for two sets of 36 randomly selected genes. *P*empirical denotes the distribution of *P* values obtained by comparing the empirical FC values to the random set. Illustration suggests that FC induced by quercetin is significantly different than might be expected by chance. **b** Kernel density estimate of log FC of quercetin and the background set. Illustration suggests that quercetin tends to induce expression levels. Quercetin denotes the FC values of 36 cell-cycle genes. Background denotes the FC values of 16,308 genes (*P* = 0.02, Kolmogorov–Smirnov test)

Polyphenols are the most abundant antioxidants in the human diet, found in fruit, vegetables, nuts and tea. They have been proposed to exert beneficial effects in a multitude of disease states, including cancer, cardiovascular disease, and neurodegenerative disorders [24]. Quercetin is one of the most abundant polyphenols and although its anti-inflammatory and anticancer properties have been well studied, much uncertainty surrounds its mechanism of action. There is an overall tendency to explain the beneficial effect of a polyphenol via its antioxidant activity, even if polyphenol concentrations in the blood barely reach the level needed for such activity (10–100 μM). Even though the exact mechanisms by which quercetin exerts its beneficial effects remain unclear, it is known to influence intracellular redox status, modulate gene expression of enzymes associated with biotransformation—such as phase I and phase II metabolism, suppress cell proliferation by interacting with type II estrogen-binding sites, inhibit chromosomal alterations and tyrosine kinase, modulate several signal-transduction pathways through actions at MEK/ERK and Nrf2/keap1, and stabilize the tumor suppressor p53 at both the mRNA and protein levels to reactivate p53-dependent cell-cycle arrest and apoptosis. The massive involvement of quercetin in cell activity requires deep investigation into its mechanism of action. This work makes use of whole-genome methods to reveal new mechanisms by which quercetin might affect cellular function. We performed Chem-seq and RNA-seq analyses on monocyte cells. Monocytes were chosen because of the ample literature on the effects of quercetin on signal transduction and gene expression (mostly anti-inflammatory and antibacterial genes) in monocytes and macrophages [25, 26]. To examine direct binding sites of quercetin to the genome via the Chem-seq method, biotinylated quercetin was synthesized and evaluated (Fig. 1, Additional file 1: Figure S1). Biotinylated quercetin’s effect on cellular gene expression was examined in comparison to that of quercetin by comparing the expression levels of the 36 cell-cycle genes to the background gene expression after biotinylated quercetin induction (Table 1). The results were consistent with the gene expression obtained after quercetin-only treatment and suggested that there are no significant changes in FC value for these 36 genes between the two treatments (*P* = 0.4, *t* test). Thus, results from Chem-seq may reflect quercetin activity in the cell. Chem-seq results were then analyzed and compared to results obtained from RNA-seq to determine the differential expression of selected genes. Our results suggested that quercetin is cis-regulatory activator of specific genes following direct genome binding. In particular, those binding sites were localized to the promotor regions of 36 genes that are mostly involved in cell-cycle pathways and skeletal and kidney development (Fig. 2c). Moreover, the RNA-seq data illustrated that those 36 genes are significantly upregulated compared to the background genome (Fig. 3b, *P* = 0.02). Our list of candidate genes was consistent with the literature showing that quercetin exerts a direct, proapoptotic effect in tumor cells and may block the growth of several human cancer cell lines at different phases of the cell cycle. Both of these effects have been documented in a wide variety of cellular models as well as in animal models. Quercetin’s high toxicity to cancer cells perfectly matches the nearly complete absence of any damage in normal, nontransformed cells [27]. We also found that such regions are enriched with DNA motifs of the transcription factor family E2F (Fig. 2d) with particular similarity to the transcription factor E2F7 which is known to be involved in the S-phase of cell-cycle regulation [28]. This result is supported by the literature as quercetin is well documented to regulate, directly or indirectly, cell-cycle and anticancer genes; it inhibits tumor necrosis factor (TNF)-induced NF-κB transcription factor [29], activates the FOXO transcription factor family [30], and is involved in transcription factor Sp1 function [31]. Moreover, quercetin is known to selectively affect cancer cell proliferation, reduce cyclin D1 activity, induce G1 phase arrest and cause tumor regression by activating the mitochondrial apoptotic pathway [10]. Some of the 36 genes that are postulated to be under cis-regulation of quercetin have been previously reported. For example, the FOXO transcription factor family [30]: 2 genes on our candidate gene list belong to the FOXO transcription factors (FOXD1 and FOXJ1; FC > 2) and may be under direct regulation of quercetin. In addition, our candidate gene list includes 4 collagen genes (COL12A1, COL19A1, COL1A1 and COL2A1; FC > 3); quercetin is involved in collagen family gene regulation [32] and is central to skeletal fragility [33] and the cell cycle [34]. Moreover, our list includes 2 genes belonging to the mitogen-activated protein kinases, MAPK14 and MAPK9, FC > 10. Those genes are well known for their involvement in resistance to colon cancer, cell-cycle arrest and cell death [35, 36].

Although the precise mechanism of quercetin activity has yet to be understood, there is vast evidence in the scientific literature of this polyphenol regulating various gene families. Furthermore, quercetin is known to bind to serum and cellular proteins and to DNA [3, 10, 37].

## Conclusions

Here, we used deep-sequencing methods to capture chromatin regions bound to quercetin. We combined the data from the literature and our presented results to propose a novel mechanism by which quercetin affects specific gene expression upon binding to DNA or genome-associated proteins. Further genome-level investigation and mapping of the direct interactions of polyphenols and chromatin genome-wide could provide new insights into the mechanisms by which a small molecule influences cellular functions, and pave the way to understanding, predicting, and controlling polyphenol responses in humans.

## Methods

### Biotinylated quercetin synthesis (Fig. 1)

#### Synthesis of 2-(3,4-diacetoxyphenyl)-4-oxo-4H-chromene-3,5,7-triyl triacetate (2)

Acetic anhydride (12.5 mL, 132 mmol) was added to a solution of quercetin (1) (5 g, 16.5 mmol) in pyridine (35 mL) and stirred at room temperature. The reaction was monitored by thin-layer chromatography (TLC). After the starting material was consumed, the reaction mixture was concentrated under reduced pressure. The crude compound was purified by column chromatography on a silica gel (4:1:1 = hexane/acetone/CH_2_Cl_2_ as eluent) and was recrystallized from CH_2_Cl_2_ to give (2) as an off-white powder (4.5 g, 8.8 mmol, 54% yield).

#### Synthesis of 4-(3,5-diacetoxy-7-hydroxy-4-oxo-4H-chromen-2-yl)-1,2-phenylene diacetate (3)

A solution of imidazole (0.05 g, 0.78 mmol, 2.00 equiv.) in CH_2_Cl_2_ (5 mL) was added dropwise to a solution of (2) (0.20 g, 0.39 mmol, 1.00 equiv.) in CH_2_Cl_2_ (10 mL) at − 15 °C in an ice/acetone bath. The resulting solution was allowed to warm to room temperature and stirred for 2 days. The reaction mixture was diluted in CH_2_Cl_2_ (50 mL) and washed with 3 M aqueous HCl (3 × 50 mL). The organic layer was then dried over MgSO_4_ and filtered. The solvent was evaporated under reduced pressure. The resulting residue was purified by silica gel flash chromatography (eluent: CHCl_3_/methanol, 97:3) to give (3) as a white solid (0.16 g, 87% yield).

#### Biotinylated quercetin adduct (6)

4-(Boc-amino) butyl bromide (0.063 g, 0.25 mmol, 1.20 equiv.) and K_2_CO_3_ (0.029 g, 0.21 mmol, 1.00 equiv.) were added to a solution of (3) (0.1 g, 0.21 mmol, 1.00 equiv.) in dimethylformamide (DMF) (2 mL) under nitrogen and stirred overnight at room temperature to obtain (4). Without further purification, acetonitrile (3 mL) and 3 M aqueous HCl (3 mL) were added to the reaction mixture and the resulting solution was stirred and refluxed for 1 h. After extraction with three portions of ethyl acetate (50 mL), the water phase was lyophilized to remove the water solvent and chromatographed using preparative C-18 reversed-phase chromatography to obtain the pure 7-aminobutyl quercetin (5) as a bright yellow solid (0.02 g, 25% yield).

7-aminobutyl quercetin (5) (0.01 g, 0.027 mmol) was dissolved in DMF (0.5 mL), and the activated (+)-biotin N-hydroxysuccinimide ester (0.011 g, 0.03 mmol) was added with 10 μL triethylamine (TEA). The mixture was stirred for 2 h at room temperature and chromatographed by preparative C-18 reversed-phase chromatography to obtain biotinylated quercetin adduct (C_29_H_33_N_3_O_9_S) (6) (0.006 g, 40% yield) as a pure bright-yellow solid (one peak appeared in the HPLC chromatogram of the product, Additional file 1: Figure S1a). This product was identified as biotinylated quercetin adduct (6) by LC–MS analysis (Additional file 1: Figure S1b).

### Chem-seq

As described elsewhere [17], the Chem-seq procedure was performed as follows: exponentially growing THP 1 cells were fixed with aqueous 1% formaldehyde solution for 20 min in cell culture medium. Chemical crosslinking was terminated, cells were collected and centrifuged, and the derived pellets were washed three times with phosphate buffered saline (PBS). Cell nuclei were prepared using the EZ-Magna Chip Kit (Mercury) as proposed in the kit protocol. Cells were lysed and cell nuclei washed. Nuclei were resuspended and sonicated. Then, magnetic streptavidin dynabeads (MyOne Streptavidin T1, Invitrogen) were preincubated in PBS containing 0.5% bovine serum albumin and either 200 μM biotinylated quercetin or vehicle (DMSO) for 6 h. Polyphenol-bound beads were subsequently washed to remove unbound quercetin. Upon obtaining bead–quercetin complexes, lysates were cleared and incubated with the beads. Beads were washed and bound protein–DNA complexes were eluted. Beads were separated and supernatant was removed to a new tube. Contaminating RNA and protein were digested by addition of RNase and proteinase K, respectively, and the DNA was purified as described in the kit. Finally, purified DNA fragments were quantified by Qbit®.

### DNA library preparation

The DNA library was prepared by Magnetic Isolation Module (NEBNext®) Ultra Library Prep Kit for Illumina and quantified by Qbit®. Size and quality of the samples were examined with a bioanalyzer (Agilent Technologies).

### RNA-seq

Total RNA was extracted using an RNA extraction kit (Norgen Biotek). Poly(A) mRNA was further purified using Poly(A) mRNA NEBNext® and RNA concentration was determined using the high-sensitivity Qbit® RNA Kit (Molecular Probes). Then, an RNA library was constructed using the Ultra™ RNA library Prep Kit for Illumina (NEBNext) following the manufacturer’s instructions. Sample size and quality were examined by bioanalyzer.

### Bioinformatics analysis

#### Chem-seq mapping and peak-calling procedure

Chem-seq data of quercetin, bead treatments and input DNA were aligned to the human reference genome hg19 using Bowtie2 [38]. To explore potential binding sites of quercetin to the DNA, we used the peak-finding algorithms MACS2 [21] and the Hypergeometric Optimization of Motif Enrichment (HOMER) peak-finding program [20].

#### Exclusion of bead signals from false quercetin peaks

Streptavidin beads are known for their affinity to DNA. We therefore subtracted nonspecific, bead-derived signals from the specific and nonspecific signals obtained from quercetin-coated streptavidin beads bound to DNA. Three assays were prepared for DNA sequencing: (i) biotinylated quercetin–streptavidin beads, (ii) streptavidin beads, (iii) input DNA. The peak-calling procedure of MACS2 and HOMER was performed as follows: first, DNA alignment of the biotinylated quercetin as the treatment and streptavidin beads as the control was used to prepare a list of differential biotinylated quercetin peaks. Occasionally, peak-calling algorithms can produce “false-positive” quercetin peaks following amplification of existing bead peaks, and therefore potential false-positive peaks need to be excluded. We therefore used one more peak-calling procedure. As part of this procedure, the biotinylated quercetin bead and the streptavidin bead DNA read alignments were used as the treatment sample, while the input reads were used as the control. Following the described procedure, we selected only nonoverlapping peaks between the quercetin/beads and the beads/input peak lists. This procedure was followed for both MACS2 and HOMER algorithms and eventually produced a significant and robust peak list.

#### RNA-seq data analysis

RNA-seq data were aligned to the human reference genome hg19 using Bowtie2 [38]. Library normalization and read count assignment for each gene was calculated using the DEseq2 package [39]. Only coding genes were considered for analysis, with each gene containing a read count threshold of > 10 reads for at least one library. Our final RNA-seq dataset contained ~ 16,308 relevant genes.

## Additional file

Additional file 1: **Figure S1.** Biotinylated quercetin analysis. **a** HPLC chromatogram of the biotinylated quercetin adduct (C_29_H_33_N_3_O_9_S, Mw = 599.1937 g/mol) using an RP-18 column. One pure peak compound eluted at 8.043 min. **b** LC–MS analysis of the product using Q-TOF LC–MS with positive electrospray ionization (ESI+) method. Two peaks appeared in the m/z spectra of the product, at 600.2033 (M + H^+^) and 622.1858 (M+ Na^+^), fitting the molecular formula C_29_H_33_N_3_O_9_S with Mw = 599.1937 g/mol. (PDF 171 kb)

## Data Availability

We declare that any data and material are available upon request.

## Abbreviations

Chem–seq: Chemical affinity capture and massive parallel DNA sequencing
ChIP: Seqchromatin immunoprecipitation followed by sequencing
DMF: Dimethylformamide
DMSO: Dimethylsulfoxide
DNA: Deoxyribonucleic Acid
FC: Fold change
FDR: False discovery rate
HOMER: Hypergeometric Optimization of Motif Enrichment
HPLC: High-performance liquid chromatography
LC–MS: Liquid chromatography–mass spectrometry
NGS: Next generation sequencing
NMR: Nuclear magnetic resonance
PBS: Phosphate buffered saline
POC: Isopropyloxycarbonylmethoxy
POM: Pivaloxymethyl
RNA: Ribonucleic acid
RNA: Triethylaminex
TLC: Thin-layer chromatography
TSS: Transcription start site
