# Supplementary material for: Genome-wide localization of the polyphenol quercetin in human monocytes
Source: BMC Genomics. 2019 Jul 23;20:606. doi: 10.1186/s12864-019-5966-9 (PMC6652105; doi:10.1186/s12864-019-5966-9)
Supplement: Supplementary file 1 — Figure S1. Biotinylated quercetin analysis. a HPLC chromatogram of the biotinylated quercetin adduct (C29H33N3O9S, Mw = 599.1937 g/mol) using an RP-18 column. One pure peak compound eluted at 8.043 min. b LC–MS analysis of the product using Q-TOF LC–MS with positive electrospray ionization (ESI+) method. Two peaks appeared in the m/z spectra of the product, at 600.2033 (M + H+) and 622.1858 (M+ Na+), fitting the molecular formula C29H33N3O9S with Mw = 599.1937 g/mol. (PDF 171 kb) [file 12864_2019_5966_MOESM1_ESM.pdf]

Additional file 1

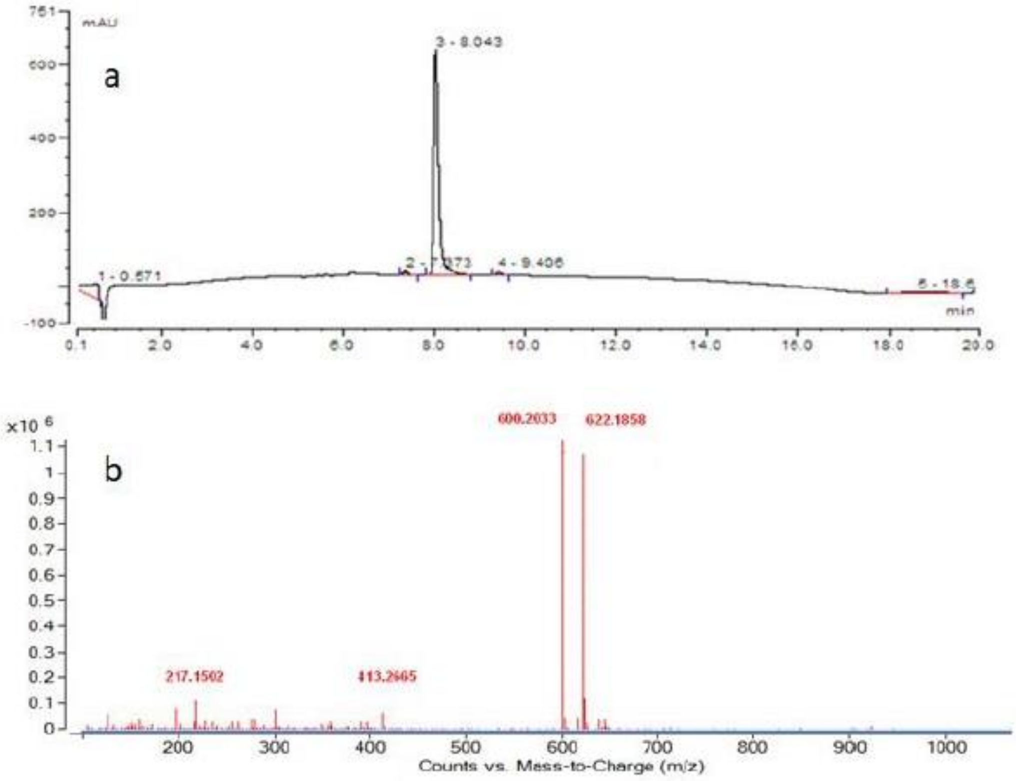

Biotinylated quercetin analysis. **a** HPLC chromatogram of the biotinylated quercetin adduct ($C_{29}H_{33}N_3O_9S$, Mw = 599.1937 g/mol) using an RP-18 column. One pure peak compound eluted at 8.043 min. **b** LC–MS analysis of the product using Q-TOF LC–MS with positive electrospray ionization (ESI+) method. Two peaks appeared in the m/z spectra of the product, at 600.2033 ($M+H^+$) and 622.1858 ($M+ Na^+$), fitting the molecular formula $C_{29}H_{33}N_3O_9S$ with Mw = 599.1937 g/mol

Abbreviations:
Chem–seq (chemical affinity capture and massive parallel DNA sequencing), ChIP-seq (chromatin immunoprecipitation followed by sequencing), Deoxyribonucleic Acid (DNA), dimethylformamide (DMF), dimethylsulfoxide (DMSO), false discovery rate (FDR), fold change (FC), high-performance liquid chromatography (HPLC), Hypergeometric Optimization of Motif Enrichment (HOMER), isopropyloxycarbonylmethoxy (POC), liquid chromatography–mass spectrometry (LC–MS), next generation sequencing (NGS), nuclear magnetic resonance (NMR), phosphate buffered saline (PBS), pivaloxymethyl (POM), Ribonucleic acid (RNA), triethylamine (TEA), thin-layer chromatography (TLC), transcription start site (TSS).
